# The Impact of Spatial and Temporal Dimensions of Disturbances on Ecosystem Stability

**DOI:** 10.3389/fevo.2018.00224

**Published:** 2018-12-21

**Authors:** Yuval R. Zelnik, Jean-François Arnoldi, Michel Loreau

**Affiliations:** Centre for Biodiversity Theory and Modelling, Theoretical and Experimental Ecology Station, CNRS and Paul Sabatier University, Moulis, France

**Keywords:** localized disturbance, rescue effect, bistability, return time, variability, persistence

## Abstract

Ecosystems constantly face disturbances which vary in their spatial and temporal features, yet little is known on how these features affect ecosystem recovery and persistence, i.e., ecosystem stability. We address this issue by considering three ecosystem models with different local dynamics, and ask how their stability properties depend on the spatial and temporal properties of disturbances. We measure the spatial dimension of disturbances by their spatial extent while controlling for their overall strength, and their temporal dimension by the average frequency of random disturbance events. Our models show that the return to equilibrium following a disturbance depends strongly on the disturbance’s extent, due to rescue effects mediated by dispersal. We then reveal a direct relation between the temporal variability caused by repeated disturbances and the recovery from an isolated disturbance event. Although this could suggest a trivial dependency of ecosystem response on disturbance frequency, we find that this is true only up to a frequency threshold, which depends on both the disturbance spatial features and the ecosystem dynamics. Beyond this threshold the response changes qualitatively, displaying spatial clusters of disturbed regions, causing an increase in variability, and even a system-wide collapse for ecosystems with alternative stable states. Thus, spanning the spatial dimension of disturbances is a way to probe the underlying dynamics of an ecosystem. Furthermore, considering spatial and temporal dimensions of disturbances in conjunction is necessary to predict ecosystem responses with dramatic ecological consequences, such as regime shifts or population extinction.

## 1 Introduction

Understanding the stability of ecosystems, i.e., their ability to recover and persist in the face of natural and anthropogenic disturbances, is of fundamental importance to ecology and conservation (May, 1973; Neubert and Caswell, 1997; Loreau and de Mazancourt, 2013). Ecosystems are spatially extended, comprised of multiple interacting communities in different locations, and therefore an important factor in understanding their stability is their spatial structure (Levin, 1992; Peterson et al., 1998; Wang and Loreau, 2016). However, while the influence of space on properties such as biodiversity and food web structure has been intensely investigated (Loreau et al., 2001; Chase and Leibold, 2002; Montoya and Sol, 2002; McCann et al., 2005), basic questions regarding spatial stability remain open. In particular, despite the fact that most disturbances (e.g., fires, pest outbreak, pollution runoff) are strongly heterogeneous in space, the impact of their spatial structure on stability is largely unknown. Similarly, their temporal dimension, e.g., their timespan or the frequency of their occurrence, is critical. Taken together, these dimensions span a vast space of possible disturbances that ecosystems can face (e.g., fires and storms). This, in part, explains why reaching a clear understanding of ecosystem stability has proven to be an extremely challenging endeavor.

Research on ecosystem stability has a long history in ecology, and numerous studies have investigated how various properties of disturbances affect ecosystem responses. The importance of spatial properties of disturbances, in particular, has been assessed by a few studies of regeneration dynamics under recurrent, spatially structured disturbances (Turner et al., 1993; Moloney and Levin, 1996; Fraterrigo and Rusak, 2008). These studies introduced the concept of landscape equilibrium and demonstrated how the spatial and temporal scales of disturbances can generate different stability patterns. A point not explicitly addressed in these studies, however, is the importance of rescue dynamics occurring at a regional scale when local recovery processes are too slow or fail altogether. This can occur in sufficiently connected ecosystems, following high-intensity disturbances (Foster et al., 1998; Fraterrigo and Rusak, 2008). In fact, recovery from a disturbance is a consequence of both local and regional processes. Local processes lead to recovery due to dynamics that are internal to local communities (e.g., birth and death of individuals), while regional processes lead to recovery by bringing in individuals from neighboring communities via dispersal (Turner, 1989; Leibold et al., 2004). These two processes mediate the large-scale system response to a disturbance, and their respective parts in this response is bound to strongly depend on the spatial connectivity of the system and, importantly, on the spatial structure of disturbances.

Recent work has made this relationship more explicit, by defining three distinct regimes of recovery from a single spatially heterogenous disturbance: Isolated, Rescue and Mixing (Zelnik et al., 2018). If a system is highly connected due to strong dispersal of organisms, then it is in the Mixing Regime, and the system’s behavior at large scales is essentially an extended version of a local system (Durrett and Levin, 1994). At the other extreme, if dispersal is low and hence each site acts separately with its own local dynamics, then the system is in the Isolated Regime, and its large-scale behavior is an aggregation of many independent small systems (Tilman et al., 1998; Yachi and Loreau, 1999). In between these two extremes is the Rescue Regime, where systems with intermediate connectivity show large-scale rescue dynamics due to the interaction between limited dispersal and the system’s behavior at the local scale (Peterson, 2000; Dai et al., 2013; Wang et al., 2017). For instance, in the study by Dai et al. (2013), a metapopulation of yeast exhibits a front structure which emerges due to interaction of dispersal with nonlinear local behavior of the yeast. A different example is found in the work of Wang et al. (2017), where the correlations between local bird populations, mediated by dispersal, leads to a spatial scaling law of the variability of populations across North America.

While the spatial structure of both system and disturbance plays no role in the Mixing regime, for weaker dispersal it does: in both the Isolated Regime and the Rescue Regime the spatial structure of the disturbance has significant effects as it can initiate qualitatively different responses that involve both local and regional processes (Zelnik et al., 2018). This is the case in an experimental study of a predator-prey protist system, in which local extinctions are met by rescue processes, which prevent synchronization of the regional metapopulation (Fox et al., 2017). We will therefore consider systems with intermediate dispersal, and focus on the effect of the spatial structure of disturbances as well as their temporal properties.

Quantifying the impact of disturbances amounts to defining relevant stability measures. If the disturbance is an isolated event, a natural measure to consider is the return time to the unperturbed state (May, 1973; Neubert and Caswell, 1997). On the other hand, in a regime of repeated disturbances (e.g., climatic events), measures of temporal variability are commonly used (Tilman et al., 2006). In the presence of alternative stable states, those repeated disturbances can cause a regime shift from one state to another. One well-known example is that of lake eutrophication (Carpenter, 2005) due to fertilizer runoff disturbances. Here the stability measure of interest is typically persistence, i.e., the probability that a system will remain in a desired state (Holling, 1973; Pimm, 1984). Importantly, these stability measures reflect not only the spatial and temporal properties of the disturbance, but also the dynamical features of the perturbed ecosystem. Exploring this interplay is the focus of our study, which we will address by considering three spatial ecosystem models with increasing nonlinear local dynamics, ranging from logistic growth to bistability. Under various perturbation scenarios we will measure their stability using return time, variability and persistence.

We begin by looking at the ecosystem’s recovery following a single disturbance, and show that changing the spatial structure of the disturbance reveals two basic recovery trajectories: isolated and rescue. Isolated recovery trajectories reflect the local resilience of the system, while rescue trajectories involve spatial processes, and their dominance signals the failure of local processes. We thus argue that the relationship between spatial structure and recovery contains substantial information about the local dynamics of the system, both close to and far from equilibrium. We continue by exploring the temporal axis of disturbances, and demonstrate a direct link between return time (following an isolated disturbance event) and temporal variability (under a regime of repeated disturbances). We find that for low disturbance frequency patterns of variability do not contain additional information in comparison to the patterns of return time. However, past a frequency threshold (which depends on the system’s internal dynamics) the variability patterns change. As we will argue, this signals the onset of a new dynamical regime driven by disturbances, which can lead to a regime shift—in our case a transition from a populated to a bare state (extinction).

Our work demonstrates that the spatial dimension of disturbances can be used to reveal information on the ecosystem’s internal behavior. Furthermore, our results illustrate that the conjunction of the spatial and temporal properties of disturbances may lead to unforeseen dynamical responses, with drastic ecological consequences.

## 2 Methods

### 2.1 Models

We assume for simplicity that the local community dynamics can be described by a single state variable *N* that represents the ecosystem’s local biomass density. We study the dynamics in multiple locations in space using partial differential equations. We define three different models that differ in their local dynamics but have identical dispersal across space with linear diffusion. In all models the local biomass may reach a carrying capacity *K*, so that *N* = *K* (the populated state) is a stable steady state in all three models. An additional solution exists for *N* = 0 (the bare state), with its stability properties differing among models.

The first and simplest model (LG) describes local logistic growth coupled with dispersal:

(1) $$N_{t}=rN(1-N/K)+d\nabla^{2}N,$$

where *N_t_* is the change in time of the local biomass and ∇^2^*N* is the second derivative in space of *N* (a diffusion term). Here *r* is the characteristic, local dynamical rate of growth, while the rate of spread by dispersal is governed by *d*. In this model the bare state *N* = 0 is an unstable solution. This is the classic model of population growth (Hall, 1988), shown to appropriately depict the dynamics of various biological systems, from the growth of unicellular organisms (Gause, 1934), to human populations (Marchetti et al., 1996).

The second model (AE) describes species dynamics with a strong Allee effect (Kramer et al., 2009), so that low biomass densities are not viable. Such dynamics have been found in a variety of species, ranging from the gypsy moth to woodland caribou (Kramer et al., 2009). The model reads

(2) $$N_{t}=rN(1-N/K)(N/\alpha -1)+d\nabla^{2}N,$$

where *α* is the viability threshold, i.e. the minimal amount of biomass *N* that is necessary to allow positive growth. This model has two alternative stable states (*N* = 0, *N* = *K*) and a single unstable state (*N* = *α*), and we assume that 0 < *α* < *K*. This is the simplest model for dynamics with alternative stable states, a property that has been found in many ecosystems (Scheffer, 2009), such as lakes (Carpenter, 2005) and coral reefs (Nyström et al., 2000).

Finally, our third model (SR) describes dynamics with slow recovery following intense disturbances, and stands as an intermediate between the two previous models. It will help us to clarify the distinction between strong nonlinearity and bistability. Its main feature is that while there is only one stable equilibrium at *N* = *K*, far from this equilibrium the return rate is very slow compared with the return rate close to equilibrium. This could model succession dynamics, for which the recovery following strong disturbances (e.g., clearcutting) is very slow, as it involves the successive colonization by different species, and not simply the regrowth of the disturbed species (Uhl, 1987), or a weak Allee effect, a prevalent feature in population dynamics (Kramer et al., 2009). The model is:

(3) $$N_{t}=rN(1-N/K){\left(N/K\right)}^{\gamma }+d\nabla^{2}N,$$

where *γ* controls the nonlinearity of the dynamics, such that at high values of *γ* local recovery is very slow following high-intensity disturbances.

For each model we can define a local potential (see left panels of Figure 2), such that its derivative with respect to *N* corresponds to the derivative of *N* with respect to time—i.e., the local dynamics. This means that the local dynamics follow the slope of this potential, so that the biomass density can be thought of as a ball moving from peaks to valleys in the landscape that the potential defines. In both the LG and SR models only one stable equilibrium exists, but the speed of return to the equilibrium may be much slower for low biomass density in the SR model. Two stable states exist in the AE model (the populated state and the bare state).

By rescaling time, space and biomass, we can effectively reduce the parameter space, and set *r* = 1, *d* = 1 and *K* = 1. Our results thus hold for any values of these three parameters. We set *α* = 0.4 to make sure that the AE model recovers from a single disturbance (see next subsection), and *γ* = 4 to make sure the return time far from equilibrium of the SR model is sufficiently slow. We focus on one-dimensional systems as they are simpler to analyze, but the qualitative results hold for other types of spatial structure such as two-dimensional systems (see Appendix D). We use a system size of *L* = 500, which is large enough to allow for the spatial dynamics to manifest itself (so that the system is not in the Mixing Regime Zelnik et al., 2018), with periodic boundary conditions. For a clearer illustration, in Figure 3 and Figure S2 we show snapshots of a two-dimensional system of size 200 × 200.

### 2.2 The Spatial Dimension of Disturbances

We define a disturbance as a change in the state variable that is forced on the ecosystem. We consider a pulse disturbance occurring at a given time, with its full effect being applied at that time. This assumption is appropriate for the many types of disturbances that are faster than the dynamics of the ecosystem, and lends itself to mathematical analysis. We choose a disturbance that removes biomass (reduces *N*), so that a disturbance of strength *s* will reduce the overall biomass of the ecosystem by *sK* (but any negative values of *N* will be set to 0 for consistency). Once a disturbance takes place, the ecosystem may recover to its original state, or a regime shift can occur if the ecosystem is bistable. We are interested here in stability and recovery dynamics, and therefore focus on parameter values for which a single disturbance cannot lead to a regime shift.

Since a disturbance need not occur uniformly across space, we vary the spatial extent of the disturbance *σ* while keeping its overall strength *s* constant. A disturbance is performed by choosing its locus, and removing some biomass in a domain of size *σ* centered around the locus. We can vary the spatial extent from *σ* = 1 for a uniform disturbance across space, to *σ* = *s* for a localized disturbance.

To measure recovery we use the return time *T* defined as the time needed for the ecosystem to recover 90% of the biomass lost to the disturbance. While the choice of a threshold is arbitrary, its specific value has no significant effect on the results as long as it is not too close to either 0% or 100% (which roughly correspond to reactivity and asymptotic resilience, respectively Arnoldi et al., 2016). By avoiding these extreme values, we simply emphasize the role played by the overall recovery dynamics, rather than by the system’s initial response or final convergence.

### 2.3 The Temporal Dimension of Disturbances

We consider a disturbance regime by repeatedly applying disturbances with a given average frequency *f*, over a time period *τ*. For simplicity we assume no correlation in space or in time, so that the time between disturbances is drawn from an exponential distribution with some average frequency (a Poisson process, see Appendix B for details), while the location of the disturbance’s center is drawn from a uniform distribution.

We use two measures of stability for a system that is disturbed repeatedly, i.e., variability, which measures how far the system ventures from its average value, and persistence, which measures how likely it is to move to the basin of attraction of a different equilibrium. We define variability *V* as the variance in time of the total biomass of the system, given a regime of repeated disturbances. In order to neglect the effect of transients, we calculate *V* over the last 80% of the simulations, which last for 10, 000 time steps. We define the collapse probability *C* as the probability that the system will be in the bare state at the end of a simulation, such that *C* = 0 means no chance of a system collapse, while *C* = 1 means that a collapse is certain. We use a longer simulation time (100, 000 time steps) to calculate *C* since we are interested in predicting a collapse before it occurs. For each of these calculations we run 100 simulations with different randomizations of the location and time of disturbances.

## 3 Results

### 3.1 Spanning the Spatial Dimension of Disturbances Reveals Local Ecosystem Dynamics

**Figure 1:**
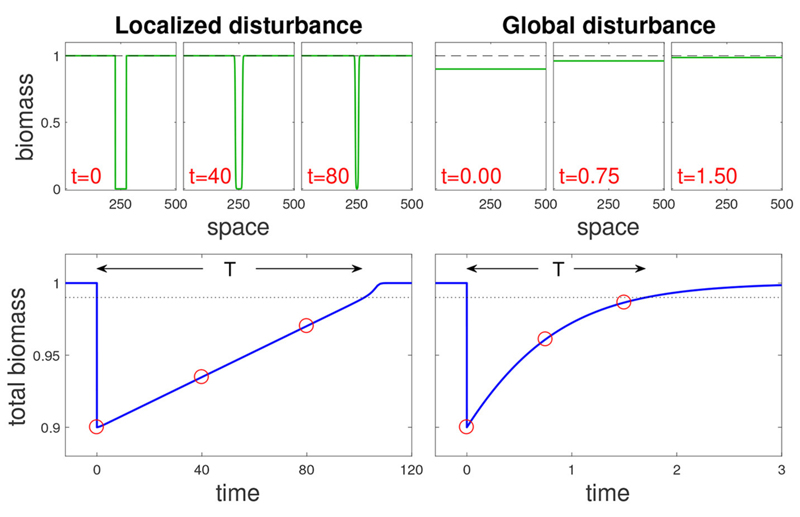
Recovery dynamics following a localized and a global disturbance (left and right panels, respectively) for the bistable AE model (see Main text). Top panels: snapshots at different times (t) along recovery trajectories, each snapshot showing a biomass spatial profile. Bottom panels show the change in overall biomass over time following the disturbance, where the dotted line denotes the threshold beyond which the system is considered to have recovered, and red circles correspond to the snapshots. Note that the return time *T* from a localized disturbance is much longer than the one from a global disturbance. Disturbance parameters are *s* = 0.1, with *σ* = 0.1 for the localized disturbance and *σ* = 1 for the global one.

We begin by looking at the response of an ecosystem to a single disturbance with varying spatial extent *σ*. We focus on disturbances with a fixed overall strength *s* = *s*_0_ for simplicity and clarity, and relax this assumption in the discussion. Thus a global disturbance *σ* = 1 (Figure 1, right panels) occurs when *N* is decreased by *s*_0_*K* in the entire system, while a localized disturbance *σ* = *s*_0_ (Figure 1, left panels) occurs when *N* is set to zero in a domain of relative size *s*_0_.

The response to a disturbance can take two possible forms: isolated recovery due to local processes, and rescue recovery due to incoming biomass from outside the disturbed region. Isolated recovery dominates the system response when each site recovers without the aid of neighboring sites (Figure 1, right panels). In contrast, rescue recovery occurs when the disturbed region cannot recover without the rest of the system, or when the bulk of the recovery occurs due to such spatial dynamics (Figure 1, left panels).

**Figure 2:**
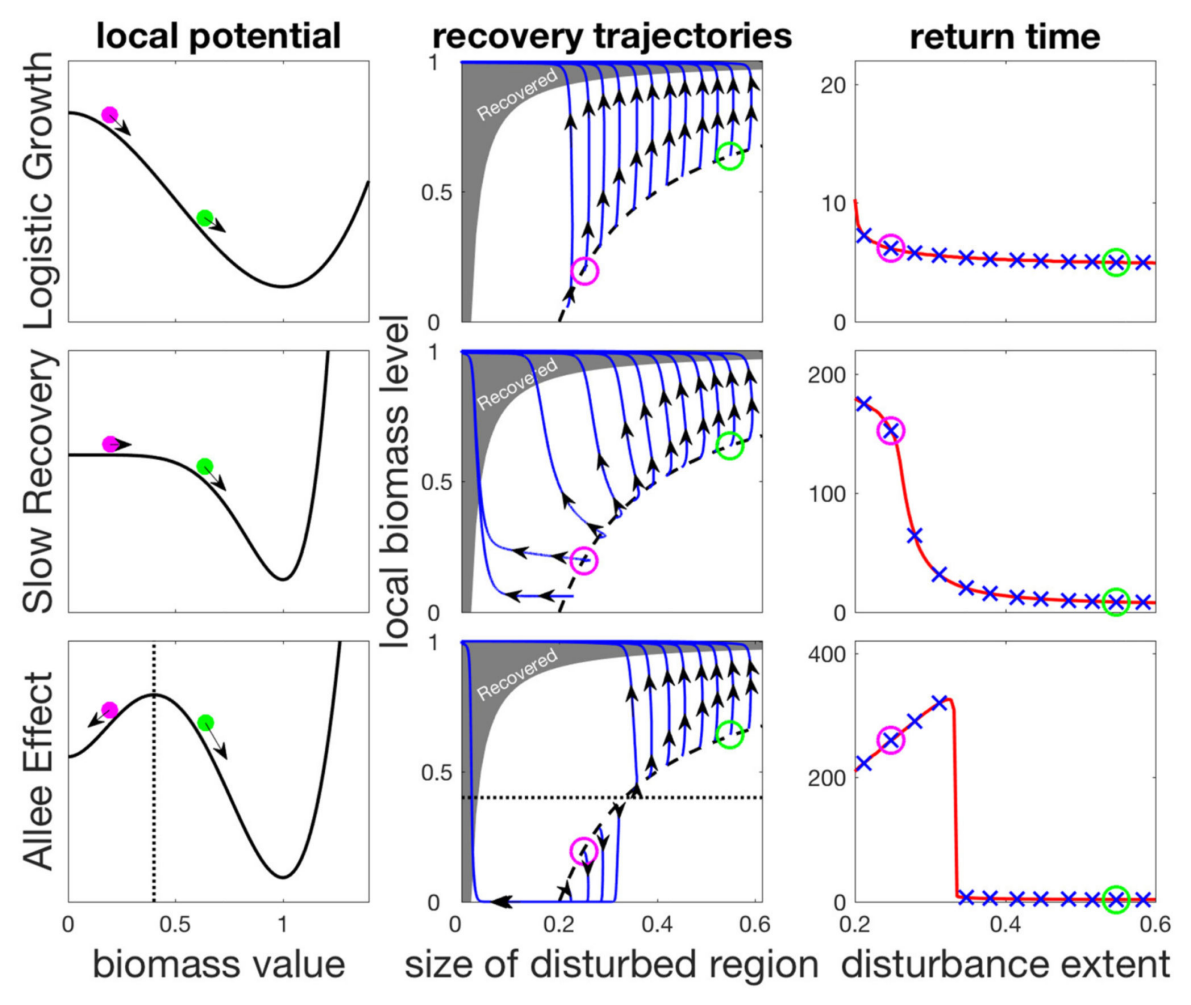
Contribution of isolated and rescue recovery as a function of disturbance spatial extent for the three models presented in the main text. The left column shows the local potentials defining local processes. Top row: Logistic (LG) model; Middle row: the highly non-linear SR model; Bottom row: bistable AE model. Middle column: isolated recovery on the y-axis, and rescue recovery on the x-axis. Black dashed line shows the equal disturbance strength used *s* = 0.2 for different disturbance extent *σ*. Blue lines are recovery trajectories, where recovery is considered complete when trajectories reach the gray shaded region. For the SR and AE model, as disturbances become more localized, a shift is observed from a dominant isolated recovery (upward trajectories) to a dominant rescue recovery (leftward trajectories), impacting return times (right column). The “x” marks in blue correspond to the different trajectories shown in middle columns. The green and magenta circles show initial states following two different disturbances (left and middle columns) and their associated return times (right column). The dotted line (bottom row) shows the local tipping point of the bistable AE model, beyond which local dynamics collapse to the bare state.

The coupling of local dynamics and dispersal results in distinct recovery processes in the three models, as shown by the trajectories in phase-space diagrams in the middle column of Figure 2. In these panels we unfold the recovery along two dimensions: the horizontal axis denotes the size of the disturbed region at a given time, while the vertical axis shows the biomass density in the disturbed region. Immediately after the disturbance, the system is along the dashed black curve, and it then changes over time until it enters the shaded region where it is considered to have recovered.

If a large part of the trajectory during recovery is horizontal, this means that the disturbed region is shrinking due to rescue recovery, which indicates a lack of local resilience, which would otherwise allow isolated recovery to take place. This behavior reflects the strong nonlinearity of local dynamics, which can be seen in the changing curvature of the local potential (Figure 2, left column). We can see that for the AE model (Figure 2, bottom row) the recovery is along a horizontal line for recovery scenarios with a sufficiently small spatial extent, so that regional processes bring about the recovery. In contrast, the recovery is entirely due to local processes in the LG model since the local dynamics are much faster here, while for the intermediate SR model a mixture of the two processes can be seen to take place.

These differences translate into markedly different values of the return time *T* (Figure 2, right panels). The vertical recovery trajectories that follow all disturbances in the LG model and large-sized disturbances in other models indicate isolated recovery, and hence small values of *T*. For the intermediate SR model localized disturbances lead to a larger contribution of rescue recovery, leading to a sigmoid shape of *T* as a function of disturbance extent *σ*. The AE model shows a similar behavior of larger *T* following localized disturbances, but the trend here shows a maximum for mid-sized disturbances. This occurs because in bistable systems, the most efficient way to perturb the system is to locally remove biomass just bellow the viability threshold, and then let the system collapse locally. Such a disturbance has an equivalent effect to that of a stronger disturbance that would remove all biomass over a larger region. The spatial recovery process will take longer to recover, thus giving larger return time values (see Appendix A for details). This explains the humped shape of return time as a function of disturbance extent. Since bistability is a sufficient condition for a hump-shaped relationship to occur, the latter could be used as an indicator of bistability. This illustrates the more general idea that considering the spatial dimension of disturbances can allow us to probe the local dynamics of a spatially extended ecosystem.

### 3.2 From Variability to Collapse Under Increasing Frequency of Disturbances

**Figure 3:**
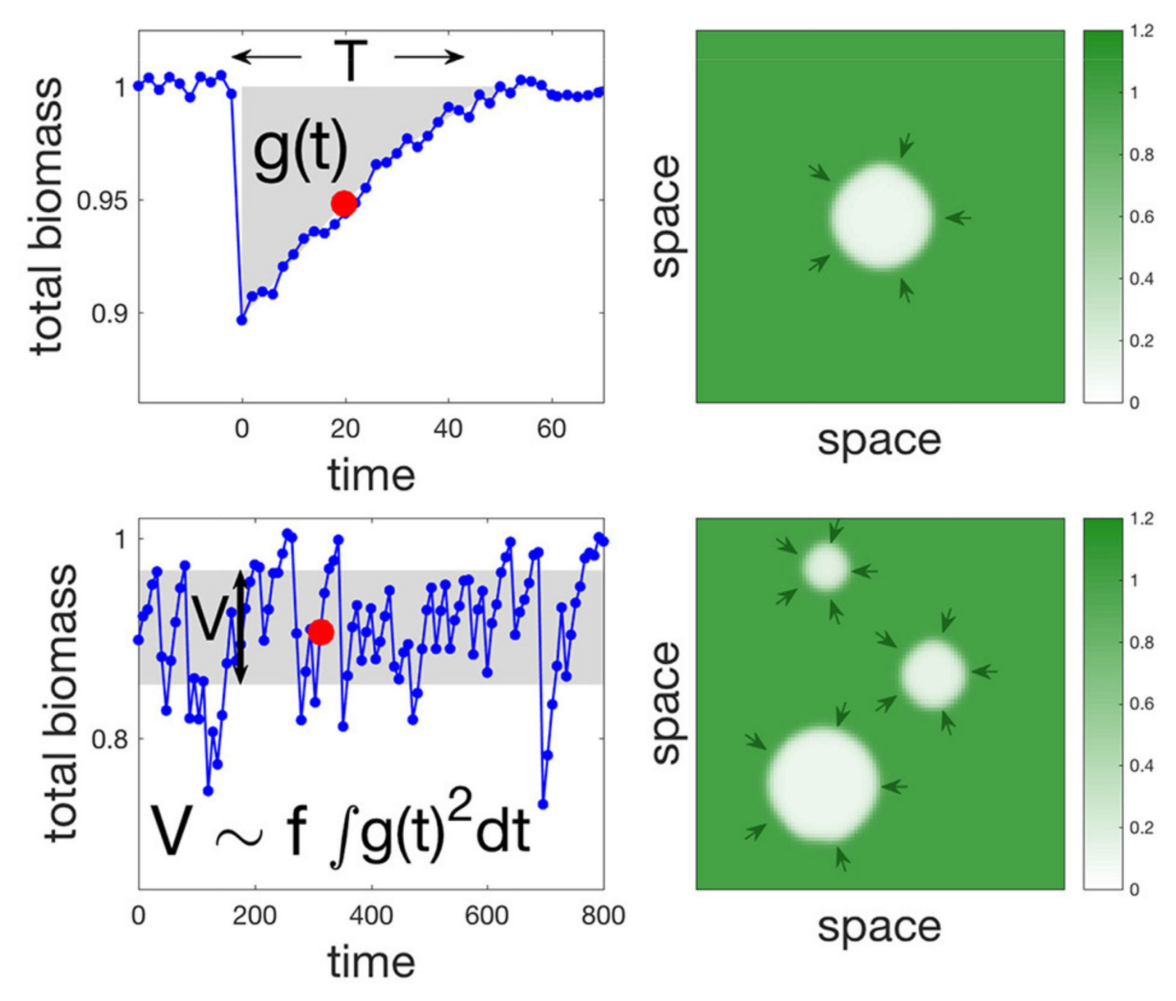
Single and multiple disturbance regimes and the relationship between return time and variability. The left panels show time series of the overall biomass, while right panels are spatial snapshots of the corresponding time-series (red dots in the left panels). The response to a single disturbance is shown in the top left panel. We focus on two of its characteristics: return time *T*, and an integral measure of the transient *g*(*t*) (see main text). The response to multiple disturbances occurring randomly at an average frequency *f* is shown in the bottom left panel. It is summarized by its variability *V* (variance of overall biomass). In the limit of low *f* there is an inherent relationship between return time and variability in the sense that *V* can be approximated by $f\int_{0}^{\infty }g^{2}(t)dt$. Simulations were made using the SR model with parameters values: *s* = 0.1, *σ* = 0.11, *f* = 0.025, and *γ* = 2. Random uniform noise was added in left panels to demonstrate how realistic time series might look like.

Natural ecosystems are constantly perturbed, leading us to consider a temporal dimension of disturbances, namely their average frequency. We therefore translate the results of the previous section on the response to a single disturbance (Figure 3, top) into an understanding of temporal variability under repeated disturbances (Figure 3, bottom). In fact, there is a direct link between the response to a single disturbance and temporal variability in response to repeated disturbances. Indeed, biomass fluctuations are the result of past disturbances, as they integrate short- to long-term responses of the ecosystem to individual disturbances (Arnoldi et al., 2016). Variability is a statistic of those fluctuations, and is therefore a function of both the integrated response to a single disturbance and the average frequency *f* of disturbances. More precisely, if *g*(*t*) traces the change in overall biomass through time following a pulse disturbance a time *t* = 0, then variability *V* can be expressed as $V=f\int_{0}^{\infty }g^{2}(t)dt$ (see Equation S15 in Appendix B). However, this identity assumes no interaction in space between the different disturbances, and therefore should not hold at high disturbance frequency.

**Figure 4:**
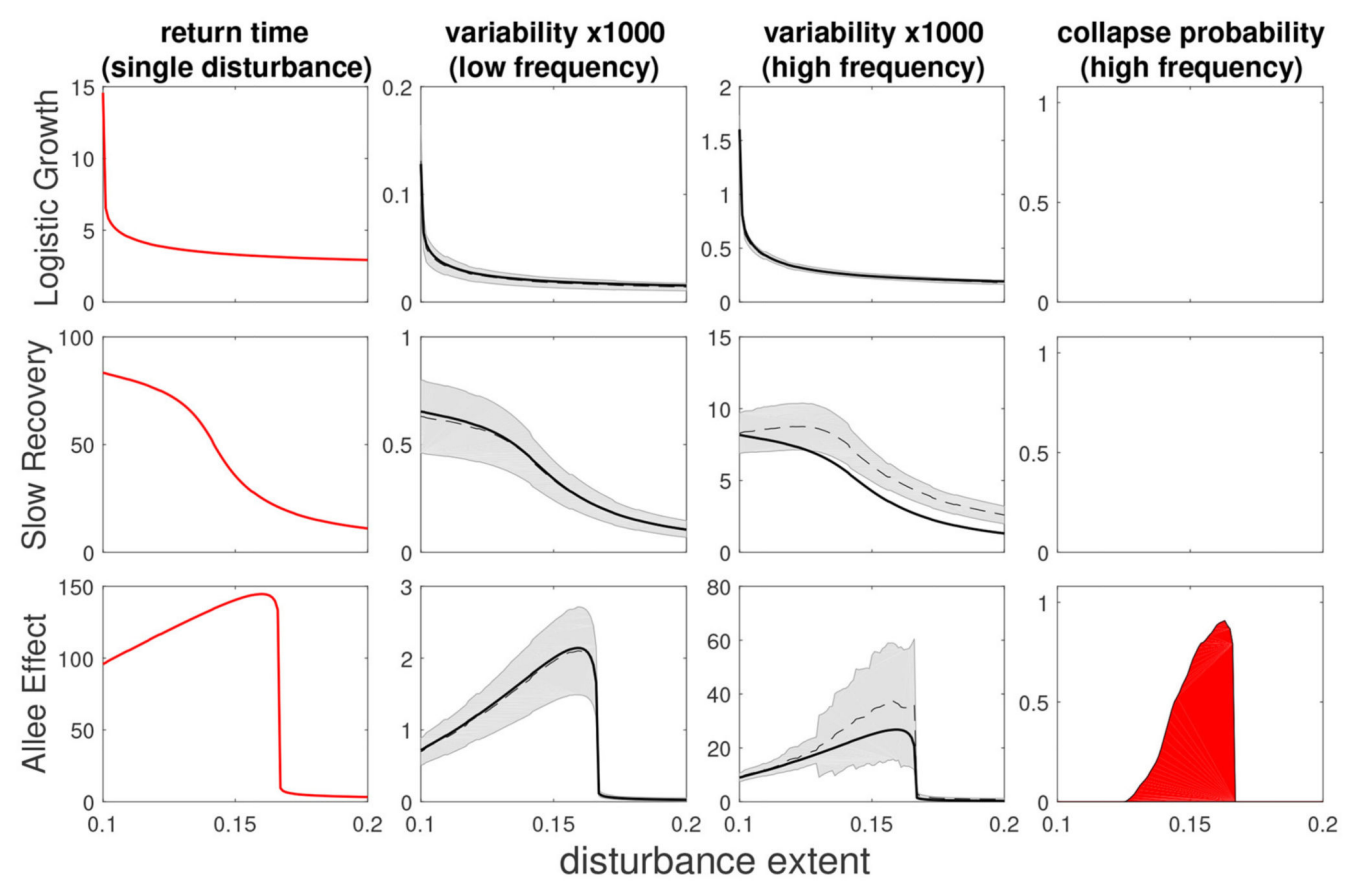
Return time, variability and collapse probability as a function of disturbance spatial extent for three models (from top to bottom: LG, SR, AE). Left column shows return times (as in Figure 2) while middle columns show variability under low and high frequency of disturbances, and right column shows collapse probability. The black dashed (solid) line is a numerical (analytical prediction) value of variability, with gray shading noting error estimation. Deviation from this prediction implies some degree of interaction between disturbances. Return time and variability are qualitatively similar with low dependency of disturbance spatial extent for the LG model but a much stronger dependency when local dynamics are highly non-linear (SR and AE models). In the case of the bistable AE model we recognize a non-monotonous “hump-shaped” dependency with disturbance extent, with mid-sized disturbances causing the most severe response. Disturbance parameters were *s* = 0.1, *σ* = 0.1, and for low frequency: *f* = 0.002, while for high frequency: *f* = 0.02.

As expected, at low frequency of disturbances the analytical approximation agrees with the numerical simulations quite well for all three models (Figure 4, second column). For higher frequencies (Figure 4, third column) where multiple disturbances often take place in the same time frame, we see a slight underestimation of the analytical approximation, although the general trend is well captured. Importantly, variability and return time show the same behavior. We see effects of regional processes on variability for more localized disturbances in both the SR and AE models, where the former shows a sigmoid shape while the latter has a hump shape, which is a consequence of the bistability in the AE model. We note that these trends hold in more general scenarios, such as disturbances with a random extent or following seasonal patterns (Appendix D).

At this point it would appear that the temporal dimension of disturbances *f* is not as informative on ecosystem behavior as the spatial dimension of disturbances *σ*. However, as *f* is increased further, a discrepancy between variability and its prediction based on recovery from a single disturbance starts to grow. This signals that the disturbances start to interact with each other, a phenomenon that is not captured by our approximation. Disturbances start to aggregate in space, which can substantially increase variability (Appendix C) due to large excursions toward low total biomass levels. For bistable systems such as the AE model, such excursions can lead to a collapse of the whole system. This is evident in the two last columns of Figure 4, in which we see, for the AE model, that the values of *σ* for which the discrepancy of variability is highest precisely corresponds to the values of *σ* for which the collapse probability is most significant. Thus, at high frequencies, disturbances of similar strength but different spatial extent lead to dramatically different responses. This example highlights the fact that the combination of spatial and temporal dimensions of disturbances can have a drastic effect on ecosystem stability.

## 4 Discussion

Investigating the role of the spatial and temporal dimensions of disturbances in ecosystem stability, we obtained four main results: (1) In comparison with a global disturbance, a localized one of the same strength can initiate a fundamentally different, and much slower, ecosystem response, especially when local dynamics are nonlinear. (2) The return time from a single disturbance and the temporal variability caused by repeated disturbances show the same trends, even for locally intense (and therefore nonlinear) disturbances. (3) The relationship between a system’s response and the spatial extent of the disturbances it experiences reveals its underlying dynamics. For instance, a hump-shaped relationship between return time and the spatial extent of the disturbances may indicate bistability. (4) The correspondence between return time and variability breaks down for high disturbance frequencies. This discrepancy signals the occurrence of spatial interactions between disturbed regions, which, in turn, may lead to a regime shift.

Although we considered simple spatially homogenous models, our results should apply to a wide range of ecosystems. Forests, savannah and shrublands might be good examples of ecosystems to which our models apply since disturbances such as fires and grazing occur frequently and are often localized, and the recovery of plant communities often follows complex succession dynamics driven by spatial processes (Adler et al., 2001; Turner, 2010; Staver and Levin, 2012). Our results, however, need not be restricted to such spatially homogeneous systems. Although we built our theory using spatially uniform models, this simplifying feature is not essential to our arguments, which only require a notion of locality. Therefore, our theory may also be relevant to less homogeneous ecosystems, such as mountain lake networks, coral reefs and riverine systems. Indeed, such ecosystems undergo different disturbances that are often strongly localized, and their dynamics may be sufficiently nonlinear (Knowlton, 1992; Campbell Grant et al., 2007; Forrest and Arnott, 2007).

Uniquely to our work, we considered systems locally pushed far from their equilibrium, and even to a different basin of attraction. In a marine ecosystem context, this could represent coral reefs (Nyström et al., 2000; Adjeroud et al., 2009) or rocky intertidal systems (Sousa, 1979; Paine and Levin, 1981), which frequently undergo intense disturbances (e.g., storm damage). These locally intense disturbances can allow rescue recovery, mediated by dispersal, to dominate the ecosystem response. In the case of the bistable (AE) model this glimpse outside the basin of attraction of the populated state is the direct cause of the hump-shaped trends of variability and return time as a function of disturbance extent. In fact, the front propagation that drives rescue recovery contains information about the ecosystem’s basins of attractions, reflecting the existence of alternative stable states and the transient dynamics between them. Thus, by observing the ecosystem’s response to localized disturbances, rescue recovery allows us to probe ecosystem dynamics far from equilibrium. For instance, comparing between different disturbed marine ecosystems may give further evidence that some have alternative states (e.g., coral reefs) while for others the dynamics show a succession process (e.g., rocky intertidal systems). This reasoning could be taken further by focusing on regions where rescue recovery takes place, e.g., analyzing the plant community structure at transition zones between grassland and forest in a savanna ecosystem (Augustine, 2003).

Spanning the spatial dimension of disturbances could thus allow us to detect nonlinearities in ecosystem behavior, revealed by the increasing local intensity of disturbances (see Figure 2). One might expect that along the temporal dimension of disturbances, increasing their average frequency could also reveal nonlinear effects, since the ecosystem becomes more strongly disturbed. In fact, increasing frequency has only a trivial linear effect, as reflected by the relation we found between return time and variability (see Figure 3). Beyond some threshold, however, a response of a different kind emerges, due to spatial interactions between disturbed regions which aggregate in potentially large-scale clusters. This causes a higher variability than expected and can, consequently, cause a global loss of persistence or a regime shift. Taking, once again, the example of corals reefs, we could ask how the impact of both natural and anthropogenic disturbances leads to a phase-shift from hard coral to fleshy algae dominance. A regime shift due to an aggregation of unrecovered regions would occur not as a typical tipping point due to loss of resilience (e.g., due to changing temperatures), but rather due to the crossing of a threshold for disturbance frequency. Importantly, in such a scenario the two dimensions, spatial and temporal, must be considered in conjunction. The threshold beyond which aggregation occurs depends strongly on the spatial extent of disturbances and hence the associated response is not a mere superposition of responses to single disturbances. In other words, this finding highlights and explains how the interplay between the spatial and temporal dimensions of disturbances can have drastic ecological consequences, such the loss of persistence. Since our findings are purely theoretical, it would be enlightening to elucidate the prevalence of this interplay in empirical systems that have undergone regime shifts (e.g., phase-shifts in coral reefs Nyström et al., 2000 or the desertification of the once green Sahara Ortiz et al., 2000).

As previously mentioned, in bistable systems the relationship between return time (as well as variability) and the spatial extent of disturbances is hump-shaped. This relation could be used as an indicator of bistability, assessed empirically by comparing time series of the same ecosystem in different regions with estimates of the intensity of single disturbances. Its implications for ecosystem management depend on the type of disturbances considered. Anthropogenic disturbances that are largely controlled, such as logging in forests (Chazdon, 2003) or large-scale fishing (Kaiser et al., 2006), can be better planned to avoid both an unpredictable yield due to high variability and an overall collapse. For many natural disturbances control is neither possible nor desired (e.g., fires in semi-arid ecosystems necessary for plant germination Wellington and Noble, 1985), but predicting their effects and the possibility of regime shifts is paramount (Kéfi et al., 2007).

**Figure 5:**
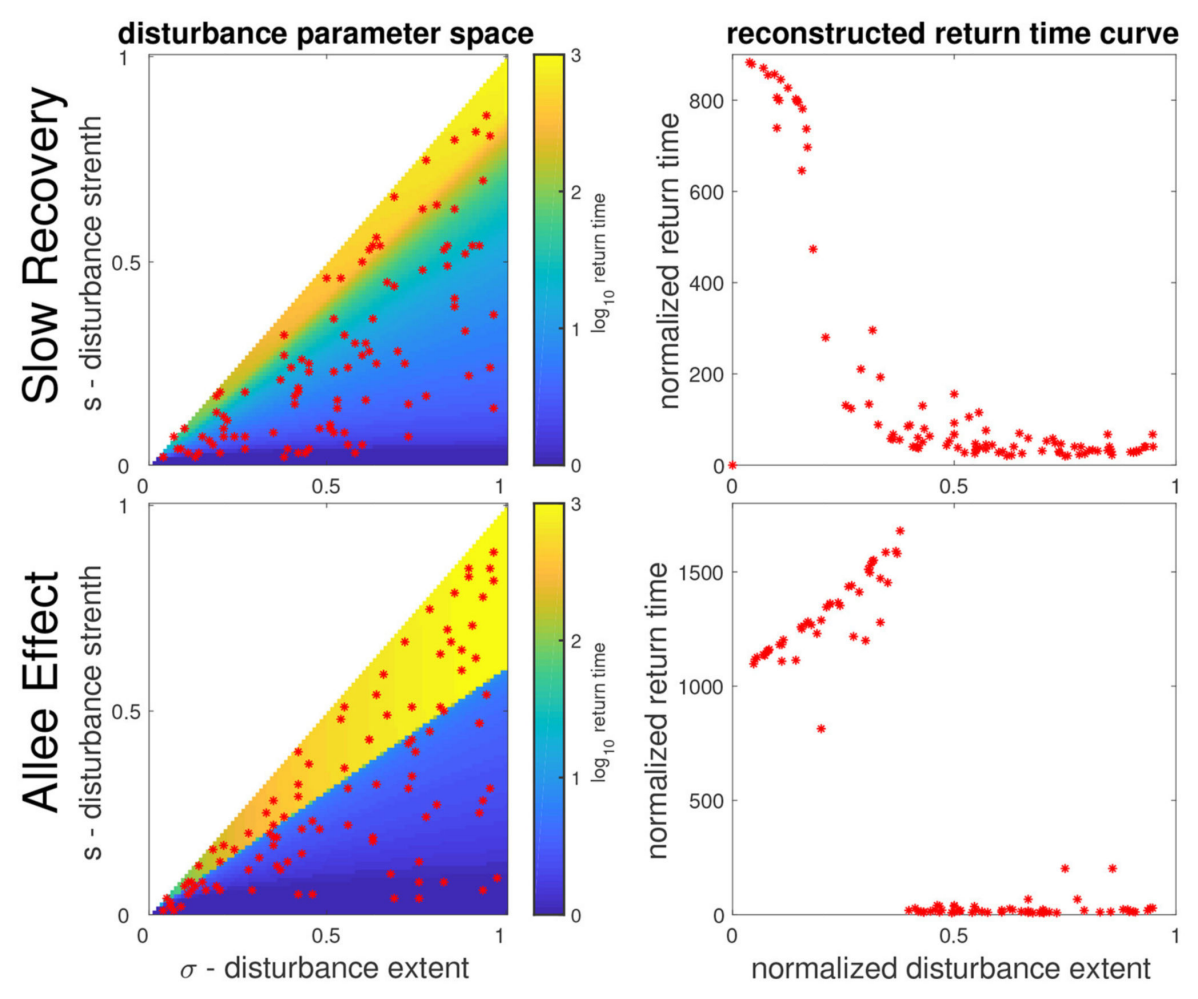
Reconstruction of return time vs. disturbance extent curve from the more general parameter space of disturbance properties. Top (bottom) panels correspond to the SR (AE) model. Left column shows the return time over the parameter space of disturbance extent *σ* (x-axis) and of disturbance strength *s* (y-axis). Right column shows the corresponding reconstruction of the return time curve, using 100 randomly chosen points (red asterisks) in the parameter space. The return time values are normalized by the disturbance strength *s*, while the normalized disturbance extent is defined as $\tilde{\sigma }=1-s/\sigma$. Note that the hump (sigmoid) shape of the curve for the AE (SR) model are easily recognizable from these reconstructions.

In order to focus on the role of the spatial properties of disturbances and allow a clearer presentation, we conducted our analysis assuming disturbances of constant overall strength. It is straightforward to extend the analysis to more general settings, such as a random extent of disturbances and seasonal patterns (see Appendix D for details). It is particularly interesting to consider the case of different values of disturbance strength *s*. As shown in Figure 5, if we randomly choose a set of points with different values of strength *s* and extent *σ*, we can use these to reconstruct a normalized version of the dependency of the different stability measures on disturbance extent. Thus we can use the different phenomena described previously, such as a hump-shape relationship as an indicator of bistability, under more general conditions, thereby making our theory more empirically accessible.

Our work is a step toward a quantitative account of spatial and temporal dimensions of disturbances, and their interplay with local and regional ecosystem dynamics. This is an important goal in the context of global change. Disturbances are of increasing frequencies and occur at different scales (which is evident, e.g., in coral reefs Jackson, 1991 and forests Turner et al., 1993), while the spatial structure of ecosystems themselves is altered by land use change, often causing fragmentation of the landscape (Harrison and Bruna, 1999). It is thus important to build a framework in which we can understand and predict the ecological impacts of this complex interplay.

## Supplementary Material

The Supplementary Material for this article can be found online at: https://www.frontiersin.org/articles/10.3389/fevo.2018.00224/full#supplementary-material

Appendices
