# Supplementary material for: The Impact of Spatial and Temporal Dimensions of Disturbances on Ecosystem Stability
Source: Front Ecol Evol. Author manuscript; Available in PMC 2019 Feb 18. (PMC6379063; doi:10.3389/fevo.2018.00224)
Supplement: Appendices [file NIHMS81667-supplement-Appendices.pdf]

Appendices of the article: The impact of spatial and temporal dimensions of disturbances on ecosystem stability

Yuval R. Zelnik, Jean-François Arnoldi, Michel Loreau

Centre for Biodiversity Theory and Modelling, Theoretical and Experimental Ecology Station, CNRS and Paul Sabatier University, 09200 Moulis, France.

Appendix A: Approximation of return time for the AE model

To understand the shape of the return time curve $T(\sigma)$ for the AE model, as seen in bottom right panel of Figure 2, we consider the initial response of the system just after the disturbance has occurred. If we ignore the effect of diffusion, then the domain that was not disturbed does not react at all, while the disturbed domain either rebounds back to the vegetated state $N = K$, or falls further to the bare state $N = 0$. This depends simply on whether the current level of biomass N_0 (immediately after the disturbance) is higher or lower than α. If we note the system size as L, the spatial extent of the disturbance as σ, and the disturbance strength (percentage of biomass cut) as s, then the biomass value at the disturbed region just after the disturbance occurred is:

$$N_0 = K(1 - s/\sigma) \tag{S1}$$

We therefore have a critical value of σ when $N_0 = \alpha$, which will decide whether the disturbed region would rebound without the effect of diffusion, as:

$$\sigma_c = \frac{sK}{K - \alpha} \tag{S2}$$

As shown in Figure S1 with a vertical red line, this approximation gives a good indication to where the return time changes behaviour rapidly. For larger disturbances $\sigma > \sigma_c$, each single point would recover from the disturbance on its own accord. Moreover, the contribution of spatial processes to this recovery would be negligible if the rate of isolated recovery r_{iso} is much faster than the rate of rescue recovery r_{res}, namely $r_{iso} \gg r_{res}$. We can use simple dimensional considerations (Meron, 2015) to estimate what are the conditions for this situation.

First, we can estimate r_{iso} by r, since it is the only parameter with units of time and no relation to space. This estimation will break down close to the unstable equilibrium (since the recovery rate will go to zero), i.e. when σ is close to σ_c. To estimate r_{res} we need to consider how fast a front between the disturbed and undisturbed domains moves. In general, the front's speed u between two domains in a model that can be written as $N_t = rf(N) + d\nabla^2 N$ would be proportional to both the rate of local dynamics r and the rate of diffusion d as $u \sim \sqrt{rd}$, due to dimensional considerations. The area of the disturbed region is σL, so that the overall time to recovery is the ratio between area and speed, $\tau \sim \sigma L/\sqrt{rd}$. We can thus estimate the rescue recovery rate as $r_{res} \sim \sqrt{rd}/(\sigma L)$, and compare this to r_{iso}. This gives us the condition $\sigma \gg \sqrt{r/d}/L$, which is easily met in the parameters we choose since L is large enough. Therefore to approximate the rate of recovery for $\sigma > \sigma_c$ we can just use the return time when we ignore spatial effects ($d = 0$). Indeed, this estimation, shown by the right-more blue curve in Figure S1, works well, even for lower values of σ close to the critical value σ_c.

For the opposite regime, $\sigma < \sigma_c$ we can easily assume that isolated recovery is negligible, since in the bistable AE model isolated recovery is not possible when locally $N < \alpha$. To estimate the rescue recovery we again consider the speed of a front between the disturbed and undisturbed domains. Here,

both of these can be assumed to be the two stable states, and under these conditions the front speed is a constant that depends only on the model parameters (Meron, 2015). In particular, the front speed u grows with the rate of local dynamics r and diffusion coefficient d as $u \sim \sqrt{rd}$, and for the AE model considered here, u also grows with α once $\alpha > K/2$ (the front is stationary at $\alpha = K/2$ due to the symmetry between the two stable states for these parameters). If we assume that once a disturbance occurs, the time until the disturbed region collapses to the alternative state is negligible, then the recovery time T in simply the time it takes the fronts to take over the disturbed region:

$$T = \frac{\sigma}{2u} \tag{S3}$$

This approximation is shown in the left-more blue curve in Figure S1. While there is a clear under-estimation of the return time in this case due to complex front dynamics, the overall trend and the value for very localized disturbances is well approximated.

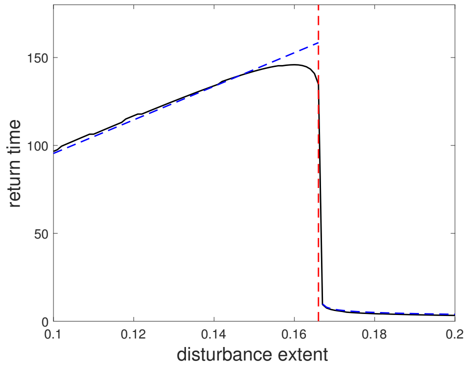

Figure S1: **Approximation of the return time for the AE model**. The black curve denotes the return time for the AE model (see Figure 2), where we set $s = 0.1$. The red vertical line shows the approximate separation point between the different regimes of recovery, $\sigma_c = \frac{sK}{K-\alpha}$. The two blue curves show the approximations of the return time in the two different regimes. These approximations are calculated as described in the text and multiplied by a factor of 0.9 since the return time (as shown in black) is defined as recovery of 90% of the biomass (see Methods).

Appendix B: Relation between return time and variability

In this appendix we derive a general relation between recovery dynamics following a single disturbance, and variability under repeated disturbances. In this manuscript we formalize disturbance events as a realization of a point process. We therefore begin by explicitly defining a point process that we use for our derivation as well as the assumptions we make for this derivation. We then detail the derivation itself, and finally we discuss the main results of this derivation.

Definitions

A point process (p.p.) Φ is a random sampling of points in $\mathbb{R}^d$ such that the number of points sampled in a bounded set is always finite (Baccelli and Blaszczyszyn, 2010). We note $\Phi(A)$ as the restriction of a p.p. to a given set A and we note $\phi(A) = \{x_1, x_2, ...\} \subset A$ as any of its realizations. Let $N(A)$ be the random variable representing the number of points in A sampled by Φ. The intensity measure Λ of Φ counts the expected number of point sampled from a given set, i.e. $\Lambda(A) = \mathbb{E}_\Phi N(A)$. Note that, if A and B are disjoint sets, $N(A)$ and $N(B)$ are independent random variables so that $\text{Cov}_\Phi(N(A), N(B)) = 0$. We call a Poisson process a p.p. such that, for any A, the mean number of points sampled is also equal to the variance so that $\mathbb{E}_\Phi N(A) = \text{Var}_\Phi N(A) = \Lambda(A)$. A homogeneous Poisson process satisfies $\Lambda(A) = f|A|$ where $|A|$ is the usual volume of A. In this case, this simply means that $\Phi(A)$ is a uniform sampling of points of A, with an expected number of points proportional to the volume of that set.

Our derivation, as given below, follows from the intuition that if the disturbances are not too frequent, so that they do not often interact in space, then we can use the superposition principle to find a relation between the response to one disturbance and the response to multiple disturbances. More concretely, our reasoning, as illustrated in Figure S2, requires the following assumptions:

1. We assume disturbance events to be realizations of a Poisson p.p. Φ in $\mathbb{R}$ of intensity measure Λ.
2. We assume no spatial interactions between disturbances, thus neglecting co-occurrence events, where multiple disturbances co-occur in a small region.

Derivation

Let us denote $g(t)$ as the recovery function from a single disturbance, so that $g(0) = sK$, where s is the relative overall strength of the disturbance, and by our definition of return time T_R we have: $g(T_R)/g(0) = 10\%$. Let $\phi([-\infty, t])$ be a random realization of all past disturbance events. The displacement time series of overall biomass under a repeated disturbance regime, $h_\phi(t) = K - N_{tot}(t)$ will be

$$h_\phi(t) = \sum_{t_k \in \phi([-\infty,t])} g(t - t_k) \tag{S4}$$

Note that for Eq.(S4) to hold, assumption (2) is necessary only if the dynamical response to an individual disturbance is non-linear. If the response is linear, a superposition principle allows Eq.(S4) to be true (Arnoldi et al., 2016), even without assumption (2). In fact, for global disturbances ($\sigma = 1$), assumption (2) cannot hold, yet Eq.(S4) still holds because a global disturbance typically induces only small displacements from K, so that the recovery is essentially linear.

By ergodicity of the point process, taking an average over long times for one realized time series is equivalent to taking a point average (say at time 0) over realizations, so that

$$\langle h \rangle = \lim_{T\to\infty} \frac{1}{T} \int_{-T}^{0} h_\phi(t)dt = \mathbb{E}_\Phi h_\Phi(0) \tag{S5}$$

We are thus left to analyse random variables of the form $\sum_{\mathcal{T}_k \in \Phi([0,T])} g(\mathcal{T}_k)$, a realization of which is $\sum_{t_k \in \phi([0,T])} g(t_k)$ (note the change of variables $-t_k \to t_k$,

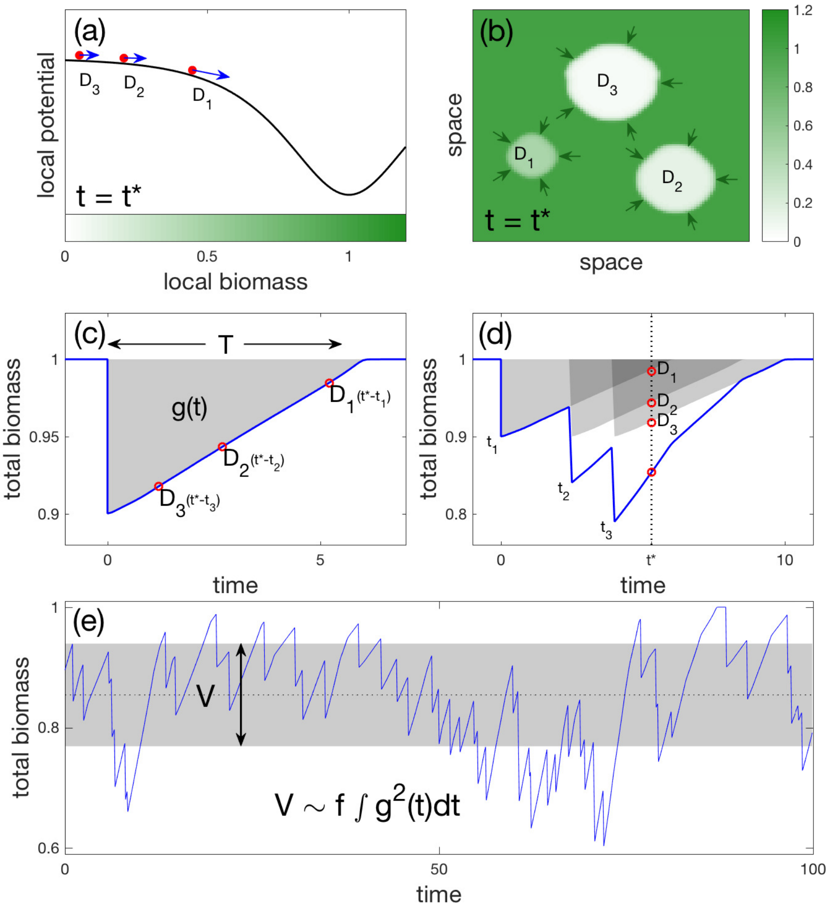

Figure S2: **Illustration of how return time following a single disturbance translates into the variability under a regime of repeated disturbances**. **(a,b)** Snapshot at time $t*$ of the local and regional dynamics, following three different disturbances ($D1$, $D2$, $D3$) at times (t_1, t_2, t_3). The local dynamics **(a)** are shown using a potential (similar to Figure 2), with three balls and arrows corresponding to the local state at each disturbed region. A snapshot of the two-dimensional system shows the regional dynamics **(b)**, as each disturbance occurred in a different location in space in a different time, and therefore has a different size and different biomass value in the disturbed region. **(c)** The trajectory of a system following a single disturbance is shown, where the current state at time $t*$ of each disturbance (if it occurred alone) is noted by a red circle. **(d)** The overall biomass over time due to the three disturbances can be approximated well by adding the three trajectories following a single disturbance in appropriate times, as shown by the grey shading. The effect at time $t*$ of each disturbance and all together is shown by the red circles. **(e)** A regime of repeated disturbances at different times results in a noisy time series (blue line) that can be measured by the variance (grey shade). This measure of variability V can be well approximated as the product of the average frequency of disturbance f and the second moment of the trajectory following a single disturbance A.

so as to restrict to positive times). We may approximate $g(t)$ with arbitrary precision by a piece-wise constant function $\tilde{g}(t) = \sum_i g_i 1_{A_i}(t)$ where the A_i's are mutually disjoint intervals such that $\bigcup A_i = [0, T]$ and the functions $1_{A_i}(t)$ are defined by $1_{A_i}(t) = 1$ when $t \in A_i$ and 0 otherwise. The numbers g_i approximate the function g in A_i. Importantly

$$\sum_{\mathcal{T}_k \in \Phi([0,T])} 1_{A_i}(\mathcal{T}_k) = N(A_i) \tag{S6}$$

From assumption (1) $\mathbb{E}_\Phi N(A_i) = \Lambda(A_i)$ where Λ is the intensity measure of Φ. We thus see that

$$\mathbb{E}_\Phi \sum_{\mathcal{T}_k \in \Phi([0,T])} \tilde{g}(\mathcal{T}_k) = \sum_i g_i \Lambda(A_i) = \int_0^T \tilde{g}(t) d\Lambda(t) \tag{S7}$$

Because the approximation of $g(t)$ by $\tilde{g}(t)$ is arbitrarily good this finally yields:

$$\langle h \rangle = \int g(t) d\Lambda(t) \tag{S8}$$

In the main text we defined variability as the variance of $h_\phi(t)$. If we repeat the above reasoning on h_ϕ^2 instead of h_ϕ we get that, with arbitrary accuracy,

$$\begin{aligned}
\mathbb{E}_\Phi h_\Phi^2(0) &\simeq \mathbb{E}_\Phi \textstyle\sum_{\mathcal{T}_k, \mathcal{T}_{k'} \in \Phi(\mathbb{R}_+)} \tilde{g}(\mathcal{T}_k)\tilde{g}(\mathcal{T}_{k'}) \\
&= \mathbb{E}_\Phi (\textstyle\sum_i g_i N(A_i))(\sum_j g_j N(A_j)) \\
&= (\textstyle\sum_i g_i \mathbb{E} N(A_i))^2 + \sum_{i,j} g_i g_j \mathrm{Cov}(N(A_i), N(A_j)) \qquad \text{(S9)} \\
&= (\textstyle\sum_i g_i \mathbb{E} N(A_i))^2 + \sum_i g_i^2 \mathrm{Var}\,(N(A_i)) \\
&= \left(\textstyle\int \tilde{g}(t) d\Lambda(t)\right)^2 + \int \tilde{g}(t)^2 d\Lambda(t)
\end{aligned}$$

where the last two lines follow, respectively, from the fact that $N(A_i)$ and $N(A_j)$ are independent r.v. if $i \neq j$ (no temporal correlations) and the fact that, for a Poisson process, $\mathrm{Var}\,(N(A_i)) = \mathbb{E} N(A_i) = \Lambda(A_i)$. In terms of the

integral norms $\|g\|_{\Lambda,n}^n = \int g^n(t)d\Lambda(t)$, what we have proved is that, under assumption (1) and (2)

$$\langle h^2 \rangle = \|g\|_{\Lambda,1}^2 + \|g\|_{\Lambda,2}^2 \tag{S10}$$

so that variability becomes

$$V = \langle h^2 \rangle - \langle h \rangle^2 = \|g\|_{\Lambda,2}^2 \tag{S11}$$

Discussion

In the particular case of a homogeneous Poisson process of intensity f, so that $\Lambda(A) = f|A|$ then we have that

$$V = f \times \int g(t)^2 dt \tag{S12}$$

as illustrated in Figure S2. Note that homogeneity is not essential to our arguments. For instance, if t is expressed in years, then seasonality effects could be modelled by taking,

$$d\Lambda(t) = f \times (1 + \alpha(2\cos^2(\pi t) - 1))dt \tag{S13}$$

with $0 \leq \alpha \leq 1$. What is essential is the absence of temporal correlations between disturbance events, which assumes that a disturbance does not influence the occurrence of future disturbances. This is not always true in natural systems, (e.g. a drought can make a fire more likely), but is a reasonable assumption for most climatic events.

Note that we assumed so far that all disturbances had the same spatial extent σ. This assumption can easily be relaxed by assuming σ to be a random variable, with p.d.f. $p(\sigma)$. The contributions to the overall variability of disturbances of size $\sigma' \in [\sigma, \sigma + d\sigma]$ is $dV(\sigma) = \|g_\sigma\|_{\Lambda_\sigma,2}^2 p(\sigma)d\sigma$; so that

$$V = \int_0^1 \|g_\sigma\|_{\Lambda_\sigma,2}^2 p(\sigma)d\sigma = \mathbb{E}_\sigma \|g_\sigma\|_{\Lambda_\sigma,2}^2 \tag{S14}$$

In this sense, the relationship Eq.(S14) is quite general. However, it requires assumption (2) to hold, especially for localized regimes of disturbances, for which the response will typically be highly non-linear. As we explain in the next section, departures from this prediction is the sign that disturbances are aggregating into long-lived disturbed regions, a process that increases variability and, in bistable systems, can lead to a regime shift.

Finally, it is interesting to note that had we assumed no temporal overlap between responses to disturbances (assuming a very low frequency of events, for instance) then we would have had

$$\langle h^n \rangle = \frac{1}{T}\int_{-T}^{0} h_\phi(t)^n dt = \frac{|\phi([-T,0])|}{T}\int g(t)^n dt \to_{T\to\infty} f\int g(t)^n dt \quad \text{(S15)}$$

where $|\phi([-T,0])|$ is the number of disturbance events that have occurred in $[-T,0]$. For a homogeneous p.p. and for $n=1$ we recover Eq.(S8). However, for $n=2$ we do not find Eq.(S10). This shows that assuming no temporal overlap between responses to compute V would induce an error equal to the squared mean displacement $\langle h \rangle^2$. An error that can easily be significant (but is of second order in f as the frequency of events goes to zero).

Appendix C: Aggregation of disturbances

As discussed in the Results section, the aggregation of disturbances is the main reason for the under-estimation of variability and for high collapse probability (Figure 4). We describe here how this phenomenon affects variability and collapse probability.

We define a disturbance aggregation as a situation when multiple disturbances occur within the same time frame in different locations so that effectively a large contiguous region in the system is disturbed at a given time. This aggregation typically reduces the total biomass, and more significantly, leads to an overall slow recovery when compared to a simple addition of multiple non-interacting disturbances. As shown in Figure S3, occurrence of such aggregation changes the recovery trajectory considerably (top panel), which leads to higher variability (bottom panel).

These events are more common for mid-sized disturbances, which is the reason that the under-estimation is most prominent for these values of σ. This is because an aggregation is more likely to occur when disturbances have both a longer time-span and a larger spatial extent. Larger values of σ mean that the size of each disturbed region is larger, and therefore it takes less disturbances to cover a certain region. On the other hand, more local disturbances (smaller σ) tend to lead to slower recovery (this can be quite different between models, depending on local dynamics), and hence the effect of each disturbance is retained for a longer time. Overall mid-sized disturbances are the middle-ground between these two conditions, and it is therefore there that disturbance aggregation occurs most frequently. For the specific case of a bistable system such as the AE model, mid-sized disturbances also have the slowest recovery, so the effect is even stronger.

We note that for bistable systems, a collapse to the alternative state is possible if enough disturbances push the system to the other state. This is essentially an extreme case of disturbance aggregation, where the disturbances cover the entire system. Therefore, when increasing the frequency of disturbances we first see a collapse occurring for mid-sized disturbances, and only for higher frequencies do we see it for smaller and larger extent of disturbances (see Figure S4).

Appendix D: Generalization of results

The results presented in this paper have focused on a simple model setup for the sake of clarity, but they can be easily generalized in various settings. For example, in eq. S13 in Appendix C we show how temporal seasonality can be taken into account and give the same overall results. We further demonstrate here that our results are not specific to a particular spatial structure, with two specific examples. We compare between one-dimensional (1D) and two-dimensional (2D) systems, looking at the results for return time, variability and collapse probability. We also consider a regime of repeated disturbances where the size of disturbances is not the same every time, but rather taken from a probability distribution.

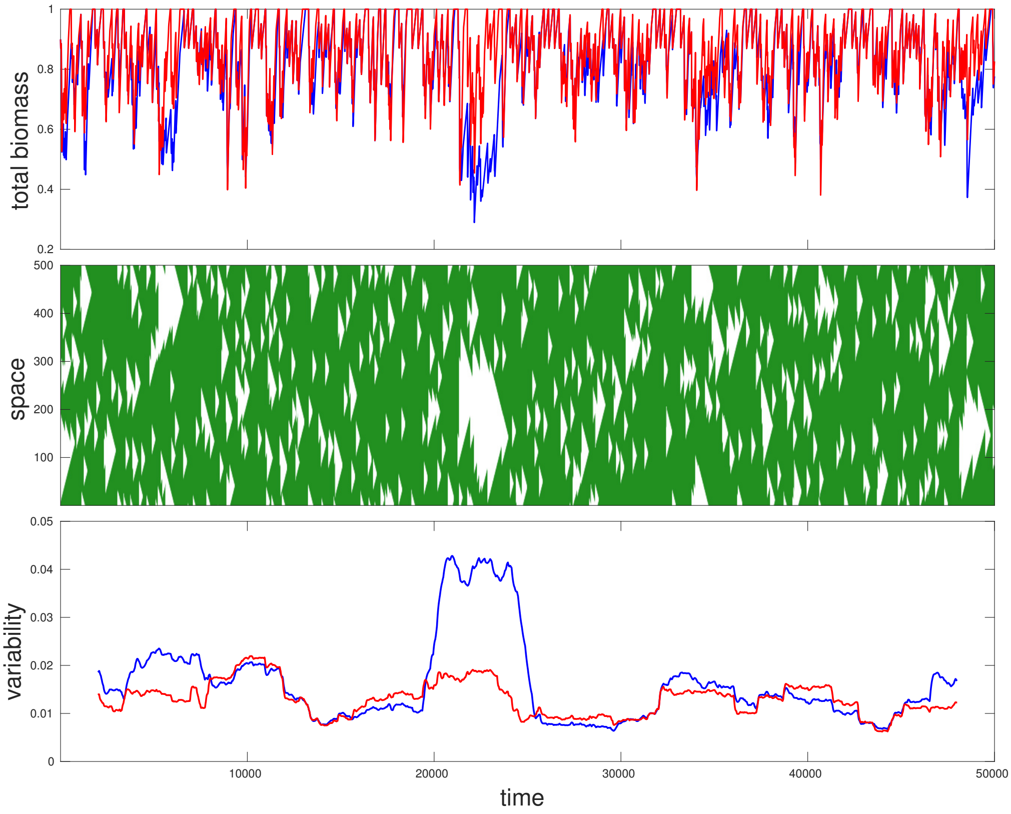

Figure S3: **Dynamics of single simulation with multiple disturbances, demonstrating the effect of disturbance aggregation.** The dynamics of a single simulation of the AE model with disturbance frequency of $f = 0.01$ and mid-sized disturbances $\sigma = 0.15$ are shown in the three panels. This is compared with a "constructed time-series" where the effects of a single disturbance are added multiple times to form a time-series that behaves as a simulation where disturbances do not interact in space (so that their effect is always linearly additive). The top panel shows the overall biomass over time, for both the actual simulation (blue) and the constructed time-series (red). The middle panel shows a space-time simulation where the vertical axis shows the space of a one-dimensional simulation, and colour depicts the amount of biomass (darker green denotes more biomass). The bottom panel shows variability calculated over a running time-window of $\tau_v = 4000$, where for each point shown, the variability was calculated for part of the time-series with length τ_v that is centred on that time point. The variability of the actual simulation is shown in blue, whereas the variability for the constructed time-series is shown in red. A large disturbance aggregation is seen starting $t = 20000$, which lowers the biomass and increases the variability considerably (blue), with no counterpart for the constructed time-series (red). Parameters used: $r = 0.5$, $s = 0.1$.

To demonstrate that the specific spatial structure of the ecosystem is not a fundamental part of our theory, we show here results for a two dimensional (2D)

ecosystem, and compare them to results for a one dimensional ecosystem (1D) that we show in the main text. For the 2D case we consider an ecosystem with the size of 200×200 with periodic boundary conditions, and use disturbances with a circular shape. As can be seen in Figure S5, the qualitative properties for return time, variability and collapse probability, as described in the main text, are the same for the 1D and 2D ecosystems. Note that the specific frequencies that are compared are not the same, but rather chosen to show the similarity in trends between the 1D and 2D ecosystems.

The trends of variability and collapse probability as described in the main text hold under more general conditions. We consider here a more realistic scenario by allowing disturbance's size to randomly vary around a fixed average. As an example we set σ to be randomly chosen from a Gaussian distribution with a standard deviation of 0.05 around a given average (Figure S6). Both the variability and collapse probability show a hump-shaped relationship with the spatial extent of the disturbance in the bistable AE model (top panels), while for models with a single equilibrium such as the SR model, no such behaviour is seen (bottom panels).

References

Arnoldi, J.-F., Loreau, M., and Haegeman, B. (2016). Resilience, reactivity and variability: A mathematical comparison of ecological stability measures. *J. Theor. Biol.* 389, 47–59. doi:10.1016/j.jtbi.2015.10.012

Baccelli, F. and Blaszczyszyn, B. (2010). Stochastic geometry and wireless networks: Volume I Theory. *Found. Trends. Network.* 3, 249–449. doi: 10.1561/1300000006

Meron, E. (2015). *Nonlinear physics of ecosystems* (CRC Press)

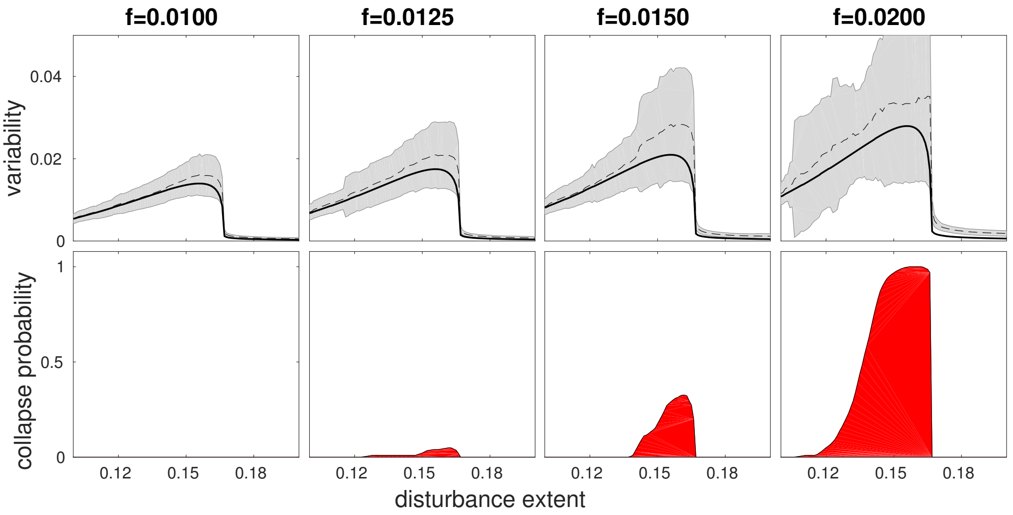

Figure S4: **Variability V (top) and collapse probability C (bottom) in the bistable AE model, for increasing frequencies of disturbances $f = \{0.01, 0.0125, 0.015, 0.02\}$.** The variability calculated from simulations is noted with a dashed black line, with grey shading noting the error estimation of the standard deviation, while the analytical approximation (Appendix B) for it is shown with a solid black line. Parameters used: $r = 0.5$, $s = 0.1$.

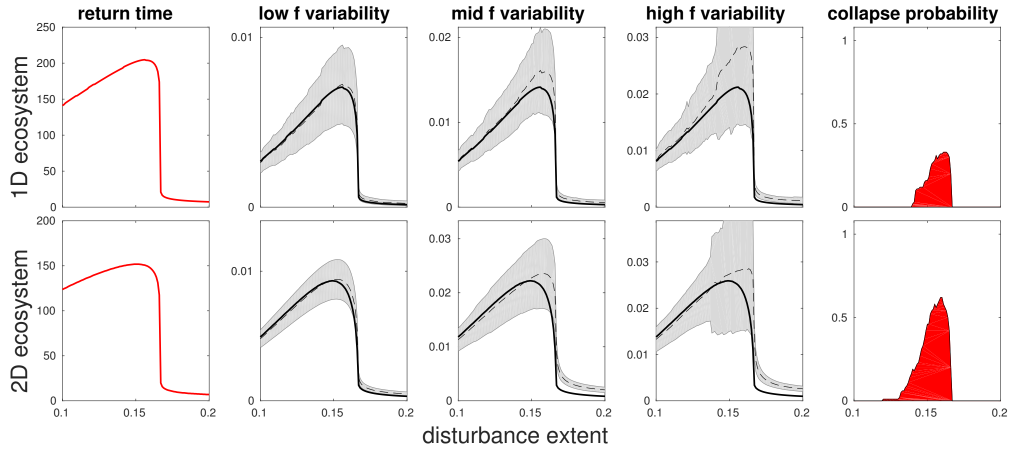

Figure S5: **Comparison of return time, variability and collapse probability between 1D (top) and 2D (bottom) ecosystems of the AE model.** The left-most column show the return time, the three middle columns show variability for different frequencies, and the right most column shows collapse probability for the highest frequency. The values of f are chosen to show similar behavior between the 1D and 2D case ($f = 0.005, 0.01, 0.02$ for 1D ecosystems, $f = 0.01, 0.02, 0.05$ for 2D ecosystems). In the middle columns the dashed line shows numerical values of variability with grey shading noting error estimation. The analytical prediction (based on the response to a single disturbance) is shown in a solid black line, where deviation from this prediction implies some degree of interaction between disturbances. Parameters used: $r = 0.5$, $s = 0.1$. 2D system is of size 200x200.

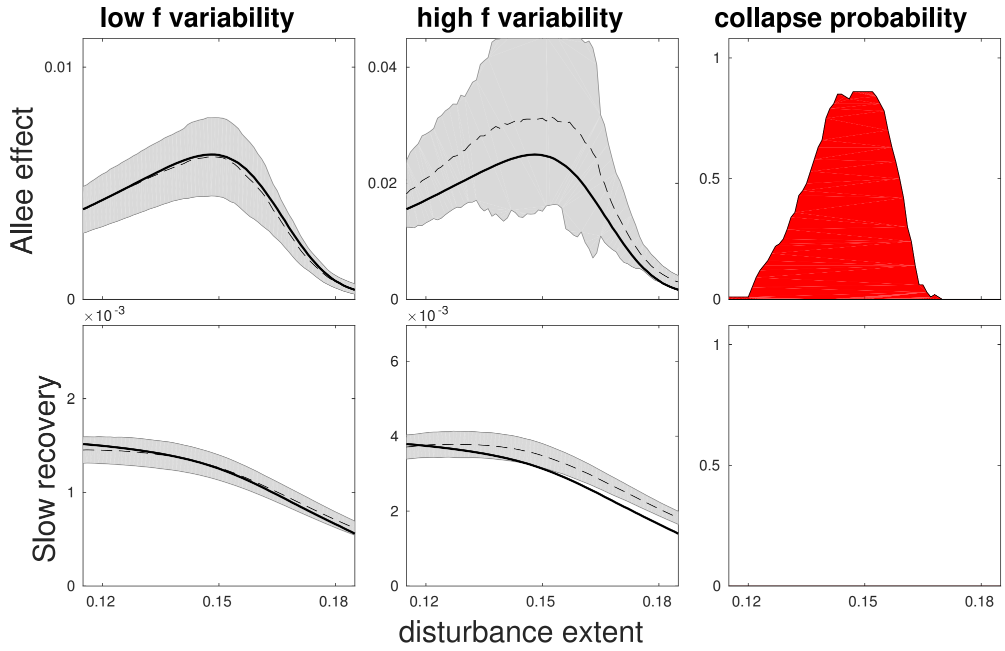

Figure S6: **Variability and the probability of collapse under a repeated disturbances with randomly distributed spatial extent.** Results are for the locally bistable AE model (top panel) and the SR model (bottom panel) that has a single equilibrium (compare to Figure 4). Left and middle columns show variability (for low and high frequency respectively), where the dashed line shows numerical estimations with grey shading noting error estimation. The analytical prediction (based on the response to a single disturbance) is shown in a solid black line, where deviation from this prediction implies some degree of interaction between disturbances. Right column shows collapse probability for the high frequency of disturbances. Disturbance extent is taken from a Gaussian distribution with a standard deviation of 0.01, where the mean is changed along the x-axis between 0.1 and 0.2. Comparing with Figure 4 we see that allowing random variations in disturbance extent leads to variability and collapse probability that are more of a smooth function of average disturbance extent while preserving the hump shape for the bistable AE model. Low and high frequencies used are $f = 0.005, 0.02$ for the AE model and $f = 0.02, 0.05$ for the SR model. Parameters used: $r = 0.5$, $s = 0.1$.
